# Lipid Structure Matters in Lysosomal Storage Disease

**DOI:** 10.1016/j.jlr.2023.100476

**Published:** 2023-11-14

**Authors:** Roger Sandhoff

**Affiliations:** Lipid Pathobiochemistry Group, German Cancer Research Center Heidelberg, Heidelberg, Germany

Ganglioside GM1 (precisely GM1a) is a major lipid component of neuronal plasma membranes, especially enriched in synaptic membranes. GM1 is involved in many functions at the plasma membrane and intracellular loci including ion transport, neuronal differentiation, G protein-coupled receptors (GPCRs), immune system reactivities, and neuroprotective signaling (1). GM1 is also an intermediate in the turnover of the other major gangliosides of brain (Fig. 1).

**Fig. 1:**
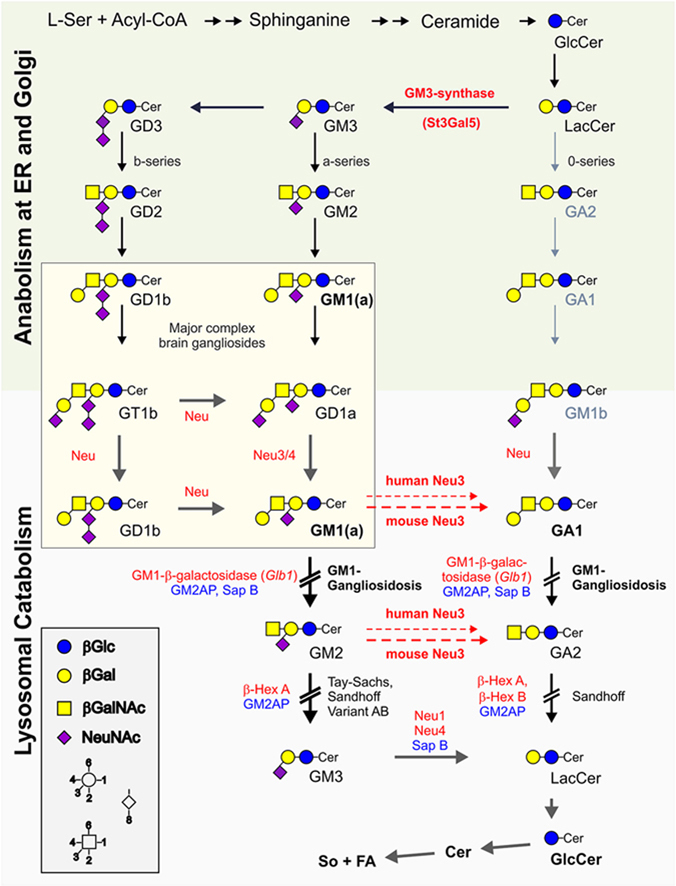
Shortened mammalian glycosphingolipid anabolism and lysosomal catabolism with a focus on major complex brain ganglioside turnover. Enzymes and corresponding activator proteins, which are relevant for the discussion, are printed in red and blue, respectively. Human diseases associated with the discussed metabolic blocks are printed in black. Major complex gangliosides of adult brain are boxed and belong to the a- and b-series. The pathway of 0-series gangliosides, indicated with gray arrows, is getting a major pathway only in GM3-synthase deficient mice. Cer, ceramide; ER, endoplasmic reticulum; FA, fatty acid; Gal; galactose; Glc, glucose; GalNAc, N-acetyl galactosamine; L-Ser, L-serine; NeuNAc, N-acetyl neuraminic acid (sialic acid); So, sphingosine.

The glycan moiety of GM1 is assembled step-wise by a group of membrane-bound glycosyltransferases using activated monosaccharide building blocks en route through the Golgi apparatus. From there, vesicular transport delivers GM1 to the outer layer of the plasma membrane. GM1 disassembly occurs after endocytosis and fusion with lysosomes, where the gross turnover of the water-insoluble GM1 is facilitated on the surface of intralysosomal vesicles. In a stepwise manner, single monosaccharides are split of the glycan moiety starting at the hydrophilic non-reducing end of GM1 (Fig. 1). In the first step, GM1-beta-galactosidase (encoded by *GLB1*) catalyzes cleavage of the glycosidic linkage of the terminal beta-galactose residue with cooperation from a lipid binding and transfer protein, either GM2 activator protein (GM2AP) or sphingolipid activator protein B (SAP-B) (2) to reach physiological turnover rates. Mutations in the *GLB1* gene that affect GM1-beta-galactosidase activity and stability cause lysosomal accumulation of GM1 and severe neurocognitive decline, termed GM1 gangliosidosis. GM1-beta-galactosidase is also a component of a lysosomal multi-enzyme complex (b-GAL complex) together with neuraminidase 1 (NEU1) and protective protein/cathepsin A (PPCA), which is required to stabilize beta-galactosidase from proteolytic degradation within the lysosomal compartment. Loss of PPCA function will lead to loss of function of both Neu1 and beta-galactosidase causing GM1 accumulation in galactosialidosis disease (3).

Several mouse models of GM1 gangliosidosis have been established by targeting the Glb1 gene, but it has been puzzling that a complete deficiency of Glb1 in mice does not cause such a severe phenotype as the human infantile form of GM1-gangliosidosis. In this issue of the *Journal of Lipid Research*, Rick Proia and colleagues nicely demonstrate that this species difference is due to a higher sialidase Neu3 activity in mice versus humans (4). By developing and characterizing a Neu3- and Glb1-double knockout mouse model to deplete Neu3, they found a drastically earlier onset of disease in mice, which now resembles more the infantile form of GM1 gangliosidosis in humans.

## How Does Sialidase Neu3 Protect Mice From the Progressive Neurodegeneration and Shortened Lifespan?

Brains of Glb1-deficient mice mainly accumulate the asialo-form of GM1, named GA1, due to the release of the sialic acid by Neu3 (Fig. 1); whereas, the combined deficiency of both beta-galactosidase and this sialidase causes a large increase in GM1 storage at the expense of GA1. The higher GM1/GA1 ratio of the double knockout mice is comparable to human infantile GM1-gangliosidosis although the summed storage of GM1 and GA1 was comparable in both mouse models (4).

## Why Do Mice Accumulate GA1 and Human Infantile GM1-gangliosidosis Patients Accumulate Primarily GM1?

One explanation experimentally given by the authors is that mouse Neu3 has a higher activity towards GM1 than human Neu3 (Fig. 1). Hence, murine Neu3 more efficiently facilitates a bypass degradation of GM1 to GA1 when the major degradation path to GM2 is blocked.

## Are These Findings Paralleling Findings of the Hexa-Deficient Mouse Model for Tay-Sachs Disease and the Hexa- and Neu3-Double Deficient Mouse Model?

Yes and no: In parallel, the phenotype of Hexa-deficient mice is much milder than that of infantile Tay-Sachs disease patients (5). These mice accumulate only minor amounts of GM2 as compared to Hexb-deficient mice, and this was ascribed to a bypass of mouse sialidases degrading GM2 to GA2 (6), which later was demonstrated to depend mainly on Neu3 (5). In contrast, instead of a single isoform of Glb1-encoded GM1-beta-galactosidase, there are 3 isoforms of lysosomal hexosaminidases, HexA (composed of an alpha-and a beta-subunits), HexB (composed of two beta subunits), and HexS (composed of two alpha subunits). Bypassing the HexA/GM2AP-dependent degradation of GM2 to GM3 with Neu3 to yield GA2 facilitates the complete degradation of GA2 by HexB in the Tay-Sachs disease mouse model (Hexa-deficiency). Therefore, GA2 is not a major storage compound in Hexa-deficient mice (6) and the sum of GA2 plus GM2 storage is much less in Hexa- as compared to Hexa- and Neu3-double knockout mice (5), and this overall reduced storage explains the milder phenotype of Hexa-deficient mice.

## Does the Type of Stored Lipid Matter?

As Proia and colleagues demonstrate here (4), there is no replacement of Glb1-encoded GM1-beta-galactosidase in the degradation of GM1 to GM2 and GA1 to GA2. This metabolic block was proposed in 1963 based on the first glycolipid analysis of a GM1 gangliosidosis patient (7). Hence, the milder phenotype of Glb1-deficient mice that have Neu3 is due to their shift in the GM1/GA1-ratio towards GA1. This suggests that the type of lipid stored does make a difference. GM1 in GM1-gangliosidosis mice has been reported to mediate activation of the unfolded protein response, which then causes neuronal death (8). Whether this is specific for the negatively charged GM1 as compared to the neutral GA1, or whether other factors play a role may require further investigation. One approach might be to introduce a GM3 synthase deficiency (9) additionally into these two mouse models. This otherwise viable deficiency blocks attachment of sialic acids to Lactosylceramide (LacCer) during biosynthesis, by that preventing the production of a- and b-series gangliosides, including that of GM1a in the mouse brain. LacCer is now routed into the 0-series gangliosides generating the still degradable GM1b (Fig. 1) (9). With a block of GM1-beta-galactosidase, these mice should only accumulate GA1, but not GM1a, even in the presence of the Neu3 deficiency.

The findings by Proia and colleagues (4) stimulate many additional thoughts, for example: If the GM1 to GA1 ratio is determined by the speed of GM1 accumulation in patients, does the GM1/GA1 ratio decrease from infantile and juvenile to adult patients? If Neu3 activity is a patient-specific factor, might that add to the individual severity of the disease? And, as noted by Proia and colleagues (4) might manipulation of NEU3 activity be a therapeutic strategy for GM1 gangliosidosis and Tay-Sachs disease?

## Conflict of interest

The author declares that they have no conflicts of interest with the contents of this article.
